# Celiac disease diagnosis in clinical practice: ESPGHAN quality of care survey from 129 pediatric hospitals across 28 countries

**DOI:** 10.1002/jpn3.70143

**Published:** 2025-07-07

**Authors:** Anna Litwin, Thu Giang Le Thi, Nabil El‐Lababidi, Angelika Kindermann, Rouzha Pancheva, Konstantinos Gerasimidis, Cristina Campos Goncalves, Paula Crespo Escobar, Tena Niseteo, Katharina Ikrath, Sibylle Koletzko, for the Quality‐of‐Care Network

**Affiliations:** ^1^ Department of Pediatrics, Division of Pediatric Gastroenterology and Hepatology, Dr. von Hauner Children's Hospital LMU University Hospital Munich LMU Munich Munich Germany; ^2^ Stiftung Kindergesundheit, c/o Dr. von Hauner Children's Hospital LMU University Hospital Munich LMU Munich Munich Germany; ^3^ Department of Pediatrics and Inherited Metabolic Disorders, First Faculty of Medicine Charles University and General University Hospital Prague Czech Republic; ^4^ Department of Pediatric Gastroenterology, Hepatology, and Nutrition Emma Children's Hospital, Amsterdam UMC Amsterdam The Netherlands; ^5^ Department of Hygiene and Epidemiology, Faculty of Public Health Prof. Paraskev Stoyanov Medical University Varna Bulgaria; ^6^ Human Nutrition, School of Medicine University of Glasgow Glasgow United Kingdom of Great Britain and Northern Ireland; ^7^ Central Lisbon University Hospital Centre Lisbon Portugal; ^8^ i+HeALTH Strategic Research Group, Department of Health Sciences Miguel de Cervantes European University (UEMC) Valladolid Spain; ^9^ Unit of Nutrition and Obesity Hospital Recoletas Campo Grande Valladolid Spain; ^10^ Referral Center for Pediatric Gastroenterology and Nutrition Children's Hospital Zagreb Zagreb Croatia; ^11^ The European Society for Paediatric Gastroenterology, Hepatology, and Nutrition Geneva Switzerland; ^12^ Department of Pediatrics, Gastroenterology and Nutrition School of Medicine Collegium Medicum University of Warmia and Mazury Olsztyn Poland

**Keywords:** children, guidelines, histopathology, potential celiac disease, transglutaminase antibodies

## Abstract

**Objectives:**

European Society for Pediatric Gastroenterology, Hepatology, and Nutrition (ESPGHAN) guidelines recommend first‐line serology for suspected celiac disease (CeD), measuring only transglutaminase antibodies (TGA‐immunoglobulin A [IgA]) plus total IgA. If TGA‐IgA is ≥10 times the normal value, pediatric gastroenterologists (pedGI) may diagnose CeD without biopsies if autoantibodies against endomysial antibodies (EMA‐IgA) are positive in a 2nd blood sample. This Quality‐of‐Care (QoC) project benchmarked diagnostic workup in clinical practice using ESPGHAN CeD guidelines as reference.

**Methods:**

A pseudonymized survey on CeD practices was sent to 141 hospitals within the ESPGHAN QoC‐network in 28 countries.

**Results:**

Questionnaires were completed by 129/141 (91.5%) hospitals, with 121 (94%) having pedGI staff. As reasons conflicting with good QoC for CeD in their setting, responders assumed knowledge deficits among the public (57%), primary care providers (64%), non‐GI physicians (16%), and pedGIs (0%). For initial testing, 66% of physicians ordered only total IgA and TGA‐IgA, 7% did not use this combination, and 29% ordered additional serology (TGA‐IgG, EMA, antibodies against deaminated gliadin peptide, or native gliadin). Regarding conflicting results for TGA‐IgA and histopathology in IgA‐sufficient children, 61% incorrectly classified negative TGA‐IgA with Marsh 2 and 57% with Marsh 3 lesions as “potential CeD,” while 49% excluded CeD in the case of villous atrophy and negative TGA‐IgA. Routine practice did not align with the ESPGHAN recommendations regarding performance of duodenal biopsies (27%), EMA‐testing (34%), and diagnosis of CeD in IgA‐deficient children (32%).

**Conclusions:**

We identified areas for improving QoC regarding both effectiveness and efficacy, in pediatric patients with suspected CeD, and consequently developed easy‐to‐use tools to improve guideline implementation.

## Abstract (highlights)

**What is Known:**

- The European Society for Pediatric Gastroenterology, Hepatology, and Nutrition (ESPGHAN) provides guidelines for celiac disease diagnosis in children.
- To ensure WHO Quality‐of‐Care (QoC) standards, the diagnostic process should be effective, safe, and efficient, to optimize resource use and reduce burden and costs.

**What is New:**

- The ESPGHAN QoC Survey identified gaps between clinical practice and guideline recommendations.
- Nearly one‐third of hospitals order unnecessary first‐line serological tests, hence increasing costs without improving care.
- Physicians misinterpreted different combinations of serology and histopathology results, leading to under‐ and over‐diagnosis of “celiac disease” and “potential celiac disease.”
- Pediatric gastroenterologists overestimate their knowledge compared to their true diagnostic performance.

## Graphical abstract

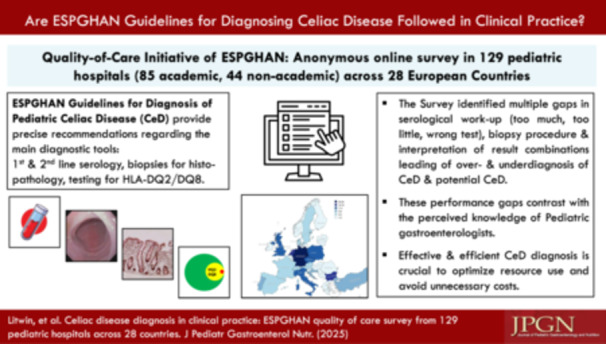

## 1 INTRODUCTION

Celiac disease (CeD) is a chronic systemic autoimmune disorder triggered by gluten. CeD is diagnosed by a combination of positive serology for autoimmune antibodies and histopathology of duodenal biopsies. Neither symptoms nor human leukocyte antigen (HLA)‐testing for the HLA DQ2 or DQ8 genetic risk markers are required as diagnostic criteria. European Society for Pediatric Gastroenterology, Hepatology, and Nutrition (ESPGHAN) guidelines recommend the two diagnostic tools: serology and histopathology^1^, ^2^ because pitfalls and contradicting test results are common in daily practice.^3^ For initial serology in children with suspected CeD, ESPGHAN recommends measuring only transglutaminase‐antibodies (TGA‐immunoglobulin A [IgA]) and total IgA, regardless of age. If total IgA is low, an immunoglobulin G (IgG)‐based test (TGA‐IgG, EMA‐IgG, or deaminated gliadin peptide [DGP]‐IgG) should be subsequently performed. High levels of TGA‐IgA, exceeding 10 times the upper limit of normal (≥10× ULN), have a positive predictive value of >99% for CeD enteropathy, which obviates the need for duodenal biopsies.^3^ Three conditions need to be fulfilled for CeD diagnosis without biopsy: the TGA‐IgA test has to meet certain criteria (e.g., linearity of calibration curve); EMA‐IgA must be positive in a second blood sample; and parents/patients should be informed of the pros and cons of the no‐biopsy diagnosis by a pediatric gastroenterologist.^2^ Biopsies are mandatory in children with low to moderate positive TGA‐IgA (>1 but <10× ULN) or IgA deficiency.^2^

Histopathology with Marsh staging cannot be considered as a standalone criterion to diagnose CeD. Interobserver variability among pathologists with respect to CeD diagnosis is high (7%–8%), insufficient size or quality of biopsies, non‐optimal fixation, and orientation can compromise accurate interpretation.^3^, ^4^, ^5^, ^6^ Conflicting results require further evaluation to confirm or exclude CeD.^2^ A common scenario is the combination of low positive (<3× ULN) or moderate (>3× to <10× ULN) TGA‐IgA levels and normal mucosal architecture (Marsh 0 or 1). After ruling out biopsy misreading, poor orientation, or inadequate sample quality, EMA‐IgA should be measured; if positive, the diagnosis of “potential CeD” is justified, requiring a different management than confirmed or excluded CeD.^2^, ^7^

In 2020, the ESPGHAN Council initiated a Quality‐of‐Care (QoC) task force and assigned three focus areas: performance of anthropometry,^8^ biliary atresia,^9^ and the diagnostic process for CeD. Before the updated ESPGHAN guidelines, a survey revealed major knowledge gaps about CeD among healthcare professionals.^10^ High QoC in their six dimensions (effectiveness, patient‐centered approach, timeliness, efficiency, and equitability) can only be ensured by continued medical research, forming and implementing strategies based on the actual state of the art (evidence‐based guidelines), as well as supporting and monitoring their translation into clinical practice.^11^

We aimed to collect comprehensive data from various hospitals across Europe to gain better insight into their current clinical practices and how these may vary between different settings and countries and align with recent guidelines.

## 2 METHODS

The QoC project's design and concept have been described previously.^8^ Briefly, the QoC Initiative conducts online surveys as a clinical service evaluation to assess the implementation of evidence‐based guidelines and current practices. Participation was voluntary; hospitals were continuously enrolled and pseudonymized. The collected data were stored in a secure platform (Castor EDC, Amsterdam, The Netherlands) compliant with the European General Data Protection Regulation.

From February 2023 to February 2024, a questionnaire survey on clinical care of children with CeD was distributed to 141 participating centers across 28 European countries within the ESPGHAN QoC Network. The questionnaire was developed by members of the QoC task force with feedback from members of the CeD Special Interest Group and the Gastroenterology Committee of ESPGHAN. Nine domains were covered, of which A to E are the focus of this manuscript:

A. *General questions* about the setting to diagnose and follow up children with CeD, and potential factors hindering the delivery of high‐QoC for affected people in their setting or their country.
B. *Serological testing for suspected or known CeD* encompassing questions on availability of resources and methods, threshold values, and indications and tools to test for serological markers.
C. *Endoscopy for duodenal biopsies and histopathology* and interpretation of different combinations with serological results.
D. *CeD diagnosis without biopsies*: practice and methods applied.
E. *Indications to test for HLA risk markers*.
F. *Documentation of confirmed diagnosis and info given to patients and caregiver*.
G. *Dietary counseling and monitoring*.
H. *Reimbursement of gluten‐free food for pediatric CeD patients.*
I. *Preference for educational materials on CeD* to improve care for their patients.

We recommended that staff members who manage GI/celiac patients in their institution, including consultants, residents, nurses, and dieticians, complete the questionnaire. Participants were instructed to select answers that most accurately reflect their current practices. Upon completing the questionnaire, participating centers received supportive educational materials translated into their preferred language.

### 2.1 Ethics statement

The data protection officer of the University Hospital of LMU Munich, Germany, reviewed the concept and questionnaires. Based on the design, the Ethics Committee granted a waiver (Project no: 20‐1150 KB).

### 2.2 Statistical analysis

Descriptive statistics were used to summarize results with counts and percentages (%). Pearson's Chi‐square test was performed to test significant differences between groups. All tests were assessed with two‐sided significance levels of 5%. Statistical analyses were conducted using SAS 9.4 (SAS Institute Inc.) and Prism 10.1.1 (GraphPad Software).

## 3 RESULTS

### 3.1 Characteristics of participating hospitals

Of 141 invited hospitals from 28 countries, 129 (91.5%) completed the questionnaire (Figure S1). Table 1 summarizes the characteristics of the participating hospitals, comparing 44 (34%) non‐academic with 85 (66%) academic hospitals.

**Table 1:** Characteristics of participating hospitals in academic versus non‐academic settings^a^ (*N* = 129).

Factors, *n* (%)	Total (*N* = 129)	Academic hospitals, *N* = 85 (66%)	Non‐academic hospitals, *N* = 44 (34%)	*p* value^b^
*General information*				
At least one consultant specialized in pediatric GI in medical team	121 (94%)	84 (99%)	37 (84%)	**<0.01**
Certified training center for pediatric GI subspecialty (*N* = 126)	81 (64%)	64 (78%)	17 (39%)	**<0.01**
Number of newly diagnosed CeD patients that are referred to the hospital per year by general pediatricians or general practitioners because of positive CeD‐specific antibodies to confirm the diagnosis (with or without biopsies)				**0.02**
None	5 (4%)	2 (2%)	3 (7%)	
1–10	26 (20%)	12 (14%)	14 (32%)	
11–30	40 (31%)	26 (31%)	14 (32%)	
31–50	30 (23%)	26 (31%)	4 (9%)	
>50	28 (22%)	19 (22%)	9 (20%)	
Number of new patients with CeD diagnosed each year in the hospital *without* positive antibodies before referral (excluding those who are referred because of positive CeD‐specific antibodies)				0.30
None	12 (9%)	7 (8%)	5 (11%)	
1–10	69 (53%)	40 (47%)	29 (66%)	
11–30	33 (26%)	26 (31%)	7 (16%)	
31–50	9 (7%)	7 (8%)	2 (5%)	
>50	6 (5%)	5 (6%)	1 (2%)	
In which setting do you see new (suspected) or known CeD patients in your hospital?				
As inpatient	57 (44%)	36 (42%)	21 (48%)	0.56
In day clinic (e.g., for endoscopy)	71 (55%)	49 (58%)	22 (50%)	0.41
As outpatient within GI‐clinic	106 (82%)	71 (84%)	35 (80%)	0.58
As outpatient within a special CeD‐clinic	24 (19%)	19 (22%)	5 (11%)	0.13
In a dietetic‐led clinic for dietary counseling	17 (13%)	14 (16%)	3 (7%)	0.12
Policy for screening for children at risk for CeD				
Siblings or children of CeD patients	118 (91%)	77 (91%)	41 (93%)	0.74
Type‐1 diabetes	124 (96%)	83 (98%)	41 (93%)	**0.03**
Down syndrome	108 (84%)	70 (82%)	38 (86%)	0.26
Williams‐Beuren syndrome	60 (47%)	40 (47%)	20 (45%)	0.50
Follow‐up patients on a regular basis after CeD diagnosis and initiation of a gluten‐free diet	125 (97%)	83 (98%)	42 (95%)	0.50
*Serological testing for suspected or known CeD*				
Test for total IgA in serum to screen for CeD	123 (95%)	83 (98%)	40 (91%)	0.08
Test for antibodies against tissue transglutaminase (TGA‐IgA)	124 (96%)	84 (99%)	40 (91%)	**0.03**
Where is the TGA‐IgA test performed? (*N* = 124)				**<0.01**
In a lab outside our institution/hospital^c^	29 (23%)	12 (14%)	17 (43%)	
In a lab within our institution/hospital	94 (76%)	72 (86%)	22 (55%)	
I do not know	1 (1%)	0	1 (3%)	
Which method is used to measure TGA‐IgA? (*N* = 124)				0.51
ELISA	50 (40%)	37 (44%)	13 (33%)	
Fluorescence Enzyme Immunoassay (EliA)	27 (22%)	19 (23%)	8 (20%)	
BioFlash	1 (1%)	1 (1%)	0 (0%)	
Chemiluminescence	12 (10%)	8 (10%)	4 (10%)	
Radioimmunoassay (RIA) or radio binding assay (RBA)	1 (1%)	1 (1%)	0 (0%)	
Other	3 (2%)	1 (1%)	2 (5%)	
I do not know	30 (24%)	17 (20%)	13 (33%)	
Which manufacturer is used to measure TGA‐IgA? (*N* = 124)				0.30
Commercial test	54 (44%)	40 (48%)	14 (35%)	
In‐house made test	1 (1%)	1 (1%)	0	
I do not know	69 (56%)	43 (51%)	26 (65%)	
Test for antibodies against tissue transglutaminase (TGA‐IgG)	82 (64%)	57 (67%)	25 (57%)	0.25
Test for antibodies against deamidated gliadin‐peptides (DGP‐IgA)	24 (19%)	15 (18%)	9 (20%)	0.70
Test for antibodies against deamidated gliadin‐peptides (DGP‐IgG)	48 (37%)	31 (36%)	17 (39%)	0.81
Test for antibodies against native gliadin (AGA‐IgA)	8 (6%)	4 (5%)	4 (9%)	0.33
Test for antibodies against native gliadin (AGA‐IgG)	8 (6%)	6 (7%)	2 (5%)	0.57
Test for antibodies against endomysium (EMA‐IgA or EMA‐IgG)	114 (88%)	78 (92%)	36 (82%)	0.09
Test is not available in our country	1 (7%)	1 (14%)	0 (0%)	
If EMA is available, where is the test performed? (*N* = 114)				**<0.01**
In a lab outside our institution/hospital^c^	42 (37%)	21 (27%)	21 (58%)	
In a lab within our institution/hospital	69 (61%)	56 (72%)	13 (36%)	
I do not know	3 (2%)	1 (1%)	2 (6%)	
What is the lowest dilution considered to be a positive result for EMA test? (*N* = 114)				0.08
1:2.5	3 (3%)	1 (1%)	2 (6%)	
1:5 (recommended in the guidelines)	15 (13%)	13 (17%)	2 (6%)	
1:10	38 (33%)	25 (32%)	13 (36%)	
1:20	5 (4%)	1 (1%)	4 (11%)	
Other	3 (3%)	2 (3%)	1 (3%)	
I do not know	50 (44%)	36 (46%)	14 (38%)	

Abbreviations: AGA, anti‐gliadin antibodies; CeD, celiac disease; DGP, deaminated gliadin peptides; ELISA, enzyme‐linked immunosorbent assay; EMA, endomysial antibodies; GI, gastrointestinal; IgA, immunoglobulin A; IgG, immunoglobulin G; TGA, transglutaminase antibodies.

^a^ Academic hospitals include university hospitals, while non‐academic hospitals encompass non‐university public pediatric hospitals, non‐academic public general hospitals with pediatric departments or divisions, church or charity‐owned hospitals, and other similar non‐university institutions.

^b^ *p* value obtained by Pearson's Chi‐square test to determine a significant difference in survey answers between academic and non‐academic hospitals. Bold *p*‐values indicate significant differences with a *p* value ≤ 0.05.

^c^ The test is performed outside of the hospital or institution, that is, in another hospital or commercial lab.

**Figure 1:**
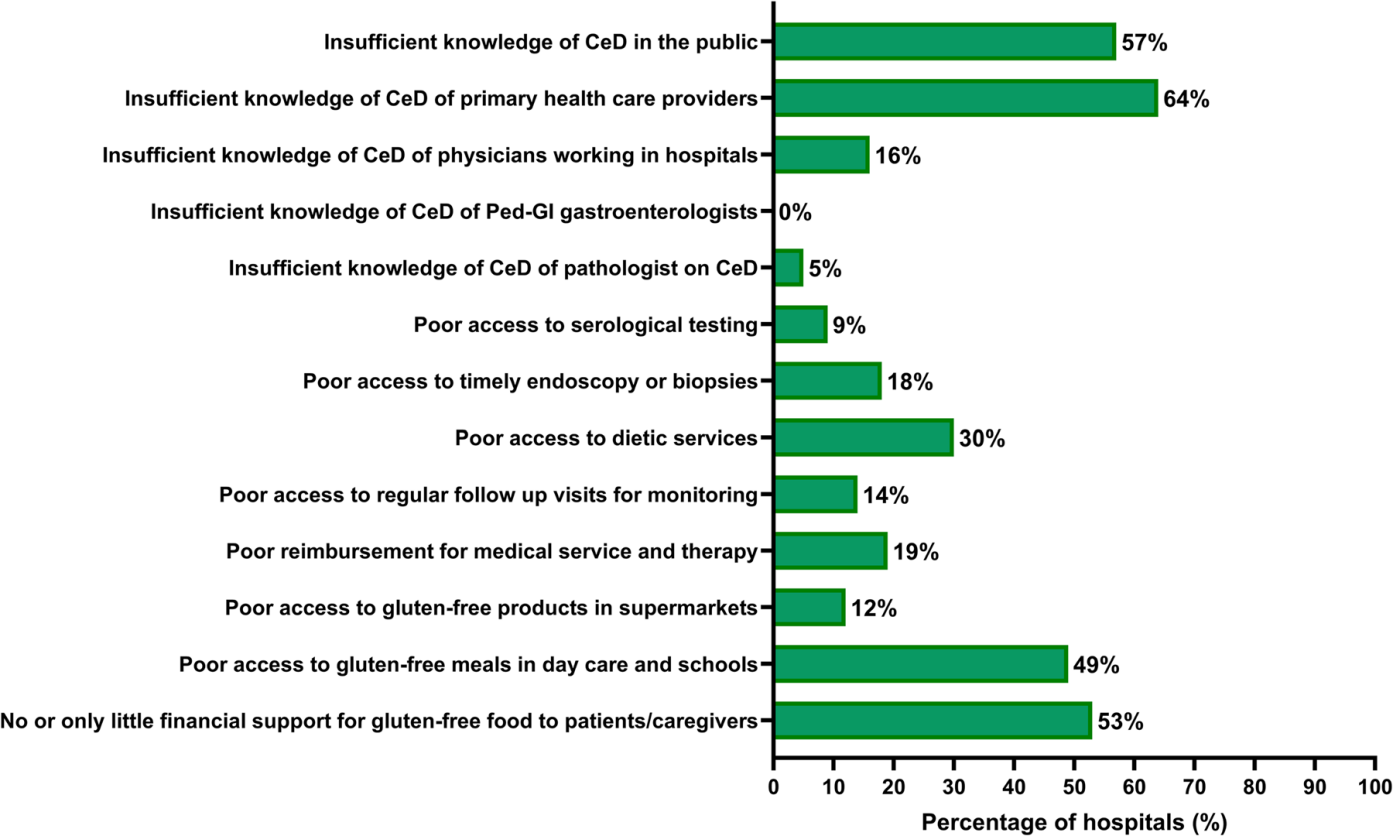
Answers to the question: “Where do you see deficits in your setting conflicting with good Quality‐of‐Care in pediatric patients with celiac disease?” (*N* = 129).

Physicians offered their opinions on the main limitations hindering good QoC in their facilities (Figure 1). Deficits in knowledge were frequently attributed to primary health care providers (64%) and public awareness (57%), while none (0%) indicated insufficient knowledge among pediatric gastroenterologists. Poor access to gluten‐free meals in day care and school was reported by 49% of participants, and insufficient financial support for gluten‐free products by 53%.

### 3.2 Serological testing

Of 129 participating hospitals, 123 (95%) ordered total IgA and 124 (96%) TGA‐IgA if CeD was suspected (Table 1). Four of the five centers that did not use TGA‐IgA did not diagnose or provide care for children with CeD. When questioned about details of the TGA‐IgA test used, 24% of participants were unaware of the method used and 56% of the manufacturer (Table 1). Contrary to the latest ESPGHAN guidelines,^2^ 24% of participants did not use TGA‐IgA for children below 2 years of age but used IgA and IgG‐based tests against DGP.

EMA‐testing was performed in 92% of academic and 82% of non‐academic hospitals (*p* = 0.09), but if measured, this was done twice as often in the laboratory within their institution in academic compared to non‐academic hospitals (72% vs. 36%, respectively, *p* < 0.01) (Table 1). Reasons for not testing (*n* = 15) include that the method is not available in their institution (*n* = 8) or country (*n* = 1), high costs for sending to an external laboratory (*n* = 4), or substitution by DGP or TGA testing in a second sample (*n* = 2). When asked about the lowest dilution considered positive, 44% did not know and another 37% reported a higher dilution than recommended 1:5 dilution.^2^ Outside of ESPGHAN guideline recommendations, EMA testing is used for initial testing in 14% of hospitals (Table S1A,B).

### 3.3 First‐line testing when suspecting CeD

In children with suspected CeD, only 66% of the hospitals strictly followed the recommendation to measure exclusively total IgA and TGA‐IgA for initial testing, in 29%, unnecessary first‐line testing was conducted (TGA‐IgG, DGP‐IgA, DPG‐IgG, anti‐gliadin antibodies [AGA]‐IgA, EMA‐IgA or IgG), while 5% used an insufficient test combination for effective case finding (total IgA without specific autoantibody test, TGA‐IgG instead, TGA‐IgA without total IgA or IgG‐based test) (Table S1A,B).

### 3.4 IgG testing in children with IgA deficiency

For second‐line testing in patients with IgA deficiency, 80% of hospitals applied at least one and 31% used two of three recommended IgG‐based tests (TGA‐IgG, DPG‐IgG, EMA‐IgG), with 6% of hospitals still using AGA‐IgG testing, which is no longer considered valuable for CeD diagnosis (Table 1).

### 3.5 Duodenal biopsies and histopathology

Eight of 129 hospitals neither performed endoscopies nor capsule biopsies. The remaining centers used endoscopy with forceps biopsies, 48% had a written protocol for biopsy collection to diagnose CeD. Table 2 depicts adherence to ESPGHAN recommendations^2^: 57% took a single biopsy per forceps, 67% collected five or more biopsies, including at least one from the bulbus, 67% separated bulbus and duodenal biopsies in different recipients, and 20% placed the biopsies on filter paper to improve orientation. Before endoscopy, 87% of the centers ensured gluten consumption; however, 20% did not routinely document this in the patient's file. The recommendation to measure TGA‐IgA at the time of endoscopy or within the last 4 weeks in their hospital/lab to allow interpretation of declining concentrations during monitoring after start of a GFD was followed by 17% and 32%, respectively.

**Table 2:** Endoscopies for duodenal biopsies and histopathology performed at participating hospitals in academic versus non‐academic settings^a^ (*N* = 129).

Factors, *n* (%)	Total (*N* = 129)	Academic hospitals, *N* = 85 (66%)	Non‐academic hospitals, *N* = 44 (34%)	*p* value^b^
How are biopsies taken for CeD diagnosis in your hospital?				**<0.01**
Forceps biopsies only	121 (94%)	84 (99%)	37 (84%)	
Neither forceps nor capsule biopsies	8 (6%)	1 (1%)	7 (16%)	
Do you or your endoscopist take single biopsies or two biopsies per forceps for CeD diagnosis? (*N* = 121)				0.78
Single biopsy at a time	69 (57%)	49 (58%)	20 (54%)	
Two biopsies at a time	50 (41%)	34 (41%)	16 (43%)	
I do not know	2 (2%)	1 (1%)	1 (3%)	
Do you have a written protocol regarding taking duodenal biopsies during upper endoscopy in patients with suspected CeD?				0.06
Yes	62 (48%)	46 (54%)	16 (36%)	
No	61 (47%)	37 (44%)	24 (55%)	
I do not know	6 (5%)	2 (2%)	4 (9%)	
What is the minimum number of biopsies taken from the duodenum (including bulbus) for CeD diagnosis?				**<0.01**
2–4	35 (27%)	28 (33%)	7 (16%)	
5 or more	86 (67%)	56 (66%)	30 (68%)	
I do not know	8 (6%)	1 (1%)	7 (16%)	
What is the minimum number of biopsies taken from the bulbus duodeni CeD diagnosis?				0.05
1	34 (27%)	24 (28%)	10 (23%)	
2 or more	86 (66%)	59 (70%)	27 (61%)	
I do not know	9 (7%)	2 (2%)	7 (16%)	
If biopsies are taken from bulbus and from pars descendens duodeni, do you put them in different transport jars?				**<0.01**
Yes, in two different jars	87 (67%)	61 (72%)	26 (60%)	
No, in the same jar	31 (24%)	22 (26%)	9 (20%)	
I do not know	11 (9%)	2 (2%)	9 (20%)	
Do you orientate the duodenal biopsies, for example, on filter paper before putting them into formaldehyde?				**<0.01**
Yes, or most times	26 (20%)	22 (26%)	4 (9%)	
No, or rarely	87 (68%)	58 (68%)	29 (66%)	
I do not know	16 (12%)	5 (6%)	11 (25%)	
Do you have the possibility in your hospital to test for TGA‐IgA deposits on frozen biopsies?				0.30
Yes	15 (12%)	12 (14%)	3 (7%)	
No	89 (69%)	59 (69%)	30 (68%)	
I do not know	25 (19%)	14 (17%)	11 (25%)	
Do you always take blood at the time of endoscopy/biopsies for TGA‐IgA (or IgG‐based test in case of IgA deficiency)?				0.13
Yes, always	22 (17%)	14 (16%)	8 (18%)	
Yes, unless measured in my hospital/lab within last 4 weeks	41 (32%)	27 (32%)	14 (32%)	
Yes, unless measured anywhere within last 4 weeks	14 (11%)	9 (11%)	5 (11%)	
Yes, but only in certain other circumstances	8 (6%)	6 (7%)	2 (5%)	
We do not have specific rules for re‐testing at time of biopsies	16 (12%)	13 (15%)	3 (7%)	
No, normally not	24 (19%)	16 (19%)	8 (18%)	
I do not know	4 (3%)	0 (0%)	4 (9%)	
Prior endoscopy/biopsies, do you assess gluten intake (with or without estimating the amount) to ensure the patient consumes sufficient gluten on a regular basis?				**0.02**
Yes, always, and the answer is documented in the file	87 (67%)	56 (66%)	31 (70%)	
Yes, but no documentation of the answer in the file	26 (20%)	19 (22%)	7 (16%)	
No, or not on a regular basis	12 (9%)	10 (12%)	2 (5%)	
I do not know	4 (3%)	0 (0%)	4 (9%)	

Abbreviations: CeD, celiac disease; ESPGHAN, European Society for Pediatric Gastroenterology, Hepatology, and Nutrition; GI, gastrointestinal; IgA, immunoglobulin A; IgG, immunoglobulin G; TGA, transglutaminase antibodies.

^a^ Academic hospitals include university hospitals, while non‐academic hospitals encompass non‐university public pediatric hospitals, non‐academic public general hospitals with pediatric departments or divisions, church or charity‐owned hospitals, and other similar non‐university institutions.

^b^ *p* value obtained by Pearson's Chi‐square test to determine a significant difference in survey answers between academic and non‐academic hospitals. Bold *p*‐values indicate significant differences with a *p*‐value ≤ 0.05.

Histology reporting includes information on Marsh staging in 92% of hospitals, crypt elongation in 89%, and intraepithelial lymphocyte count in 88%. The villous‐to‐crypt ratio and correct orientation of biopsy were reported in 57% and 60% of the hospitals, respectively.

### 3.6 Interpretation of histological and serological results for CeD diagnosis

When asked about their practices in case of Marsh 0 or 1 findings and TGA‐IgA positivity, 75% of responders contact their pathologist to review the case; 69% diagnose “potential CeD” if EMA IgA is positive; 65% repeat TGA‐IgA testing, and if confirmed positive, they diagnose “potential CeD.” If biopsy material were insufficient, 35% would repeat biopsies, while 11% would re‐biopsy in all cases with these discrepant results. Notably, 16% would diagnose “potential CeD” without any further evaluation.

Table 3 summarizes responses from 129 hospitals to the question: “In which scenario do you consider CeD diagnosis as confirmed in a child with normal total IgA considering the finding in the histopathology report and TGA‐IgA results?” We provided 12 different scenarios (a–l) and 4 options for interpretation: (i) CeD confirmed, (ii) no CeD, (iii) potential CeD if EMA is positive, or (iv) I do not know.

**Table 3:** Interpretation of the histological and serological findings for CeD diagnosis (*N* = 129).

In which scenario do you consider CeD diagnosis as confirmed in a child with normal total IgA, considering the finding in the pathology report and TGA‐IgA results? (*N* = 129)				
Histology and serology findings	Interpretation (%)			
	CeD confirmed	No CeD	Potential CeD if EMA positive	I do not know
(a) Marsh 3 and TGA positive	**95%**	0%	2%	3%
(b) Marsh 3 and TGA negative	24%	9%	57%	**9%**
(c) Marsh 3 and TGA negative and DPG‐IgG positive	34%	9%	43%	**14%**
(d) Marsh 2 and TGA positive	**80%**	1%	16%	3%
(e) Marsh 2 and TGA negative	10%	19%	61%	**10%**
(f) Marsh 2 and TGA negative and DPG‐IgG positive	19%	13%	56%	**12%**
(g) Marsh 1 and TGA positive	12%	12%	**69%**	7%
(h) Marsh 1 and TGA negative	1%	**61%**	30%	8%
(i) No atrophy and TGA negative	0%	**88%**	7%	5%
(j) Atrophy without Marsh stage and TGA positive	**26%**	8%	50%	16%
(k) Atrophy without Marsh stage and TGA negative	0%	49%	29%	**22%**
(l) Atrophy without Marsh stage and TGA negative and DPG‐IgG positive	5%	36%	33%	**26%**

*Note*: The row percentages (%) represent the proportion of physicians' interpretations in confirmed CeD, no CeD, potential CeD if EMA positive, or “I do not know” for each scenario of histology and serology findings in relation to the total 129 survey answers. Bolded results indicate the most appropriate answers according to the recommendations in the ESPGHAN guidelines.^1^ 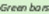 *:* Correct answer according to information provided. 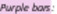 The information provided does not allow to diagnose CeD. 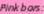 The information provided does not allow to exclude CeD diagnosis. 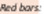 False diagnosis of “potential CeD.” 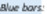 Diagnosis of “potential CeD” may be accepted, although positive TgA‐IgA is a criterion of this diagnosis.

Abbreviations: CeD, celiac disease; DGP, deaminated gliadin peptides; EMA, endomysial antibodies; GI, gastrointestinal; IgA, immunoglobulin A; IgG, immunoglobulin G; TGA, transglutaminase antibodies.

Scenario (a) Marsh 3 and TGA‐IgA positive, 95% would correctly diagnose CeD.

Scenario (b) Marsh 3 and TGA‐IgA negative: 24% would diagnose CeD, 57% potential CeD, both contradicting the guidelines.^2^ The correct option is “I do not know” because, with the given information, the scenario is unclear.

Scenario (c) is identical to scenario (b) except for additional positive DPG‐IgG: even more (34%) incorrectly confirmed CeD, ignoring that a positive DGP‐IgG result in the absence of positive IgA‐type autoantibodies (TGA or EMA) in an IgA‐competent child is insufficient for CeD diagnosis,^12^ and that DGP‐IgG is highly unspecific in very young children.^13^ Therefore, the diagnosis of scenario (c) is unclear, as in scenario (b).

Table 3 highlights frequent instances of both confirming and excluding CeD diagnoses despite insufficient information. Identical data, such as in scenarios (a) and (j) or (b) and (k), leads to varying interpretations among participants. Regarding the diagnosis of potential CeD, there is a high degree of uncertainty and inconsistent knowledge. Although only scenario (h) permits a diagnosis of potential CeD, responses ranged from 2% to 61% across other combinations.

### 3.7 Diagnosing CeD without biopsies

The ESPGHAN guidelines introduce an option to diagnose CeD without duodenal biopsies, provided the defined criteria are met. However, 15 (12%) participating hospitals did not use this approach, with reasons detailed in Table S2. Among the remaining 114 hospitals, 96% required TGA‐IgA levels ≥10× ULN, and 44% applied this option in over 80% of eligible children. Discrepancies with the guidelines are noted, with no differences between academic and non‐academic hospitals: 32% apply no‐biopsy diagnosis in IgA‐deficient children, and only 50% ensure that the TGA‐IgA test is validated for no‐biopsy use. Approximately half of the symptoms or malabsorption are required to diagnose CeD without biopsies.

### 3.8 Testing for HLA DQ2/DQ8

Access to HLA testing without justification was reported by 67% of participants, an additional 15% may use it in patients with certain conditions, and 5% have no access at all (Table S3). When asked what testing method was used, 36% reported a commercial test covering frequent alleles, and only 6% could request results of rare risk alleles if needed, but 44% did not know which method was applied. Figure S2 shows the different indications for HLA testing given by the 122 hospitals.

## 4 DISCUSSION

This QoC project surveyed current clinical practices to diagnose and manage CeD in hospitals across Europe and compared results with the current ESPGHAN guidelines.^2^ Guidelines should enable healthcare professionals to make a diagnosis with a high degree of certainty to avoid over‐ and underdiagnosis. This is particularly important for a lifelong disease such as CeD, which has a highly effective dietary treatment available. However, once on a strict GFD, confirming the diagnosis is impossible or burdensome, requiring repeated endoscopies before and during a gluten challenge.^14^ To fulfill QoC standards, the diagnostic process must be effective and safe, but efficient to maximize resource use and avoid unnecessary expenditure.^15^ For example, adding EMA‐testing to first‐line measurement of TGA‐IgA and total IgA increases costs and laboratory resources, considering that 100 individuals with unspecific symptoms like abdominal pain or constipation need to be screened to identify 2 to 4 patients with CeD.^16^, ^17^ Among the participating hospitals, 29% used one or more additional antibody tests for initial screening, including EMA and other less specific serological tests. False‐positive serological test results increase costs and induce anxiety in families, leading to unnecessary and potentially harmful follow‐up investigations.

CeD diagnosis is often perceived as straightforward; however, in practice, this is not always the case. The presence of any of the most common symptoms has a low predictive value for CeD.^17^, ^18^ HLA testing helps to rule out but not confirm CeD, and certainty of exclusion depends on the applied method.^2^ Notably, 44% of the participants did not know which method their hospital uses for HLA DQ2/DQ8 testing.

Serology and histopathology, the tools for CeD diagnosis, are both prone to pitfalls and errors. Tests to determine TGA antibodies are not standardized.^2^, ^19^ A high intertest‐ and interlab‐variability was disclosed in the ProCeDE study. Sera of 704 prospectively enrolled children were measured head‐to‐head in a central laboratory applying eight different commercial TGA‐IgA tests. Total agreement among all eight tests occurred 82% (5.1% concordant negative and 76.9% concordant positive results), but 18% showed discrepancies. This indicates that, for clinical practice, the arbitrary choice of a test may lead to false‐positive or false‐negative results in a proportion of children. Interlaboratory comparison using identical commercial TGA‐IgA assays identified a substantial number of outliers regarding titer levels.^3^ Nevertheless, high titer (>10× ULN) results were safe to predict CeD enteropathy, with four exceptions in two of the eight commercial tests.^3^ Because of these shortcomings, a pediatric gastroenterologist knowledgeable of these possible pitfalls should be involved, regardless of whether a diagnosis is made with or without biopsy.^2^

This survey identified important knowledge gaps that conflicted with high QoC. For example, to investigate children younger than 2 years of age, almost a quarter of hospitals substitute TGA‐IgA with antibodies against DGP or AGA. In healthy infants, these antibodies are frequent and transient after gluten introduction, and in the absence of positive TGA‐IgA, they have not been associated with increased risk for later CeD development.^13^

Another knowledge gap is related to the interpretation of different TGA‐IgA and histopathology result combinations in an IgA‐sufficient child. The definition of “potential CeD”^2^, ^7^ was fulfilled in only 1 of the 12 scenarios and correctly chosen by 69% of participants. However, this diagnosis was also assigned in various frequencies to the other 11 scenarios, including those reporting atrophy or Marsh 2 or 3 lesions. Table 3 also discloses that participating physicians tend to confirm or exclude CeD diagnosis despite contradicting or insufficient information. These obvious knowledge gaps contrast with the self‐perceived competence of pediatric gastroenterologists (Figure 1).

A strength of this survey was the wide range of 129 hospitals across 28 European countries, the high response rate of 91.5%, the very detailed questionnaires providing good insight into routine clinical practice in the participating institutions, and trust in the anonymity of their answers.^8^ Our survey has several limitations. Although the QoC survey was announced on the ESPGHAN website and promoted by society members and national Pediatric Gastroenterology Societies, a response bias is likely. There was an over‐representation of academic institutions (66%), with 94% of participating centers having at least one pediatric gastroenterologist on staff. Therefore, the responses of participating hospitals tend to reflect centers with greater expertise and interest in CeD. Of note, participating hospitals are not representative of their respective countries or Europe as a whole. Differences in healthcare systems and resources may impact diagnostic procedures. Conducting the survey only in English may cause misunderstanding among respondents for whom English is not the primary language.

Despite these limitations, our results contribute to a better understanding of the quality of diagnosis of children with suspected CeD living in Europe. In a subsequent publication, we will present survey results on the management of patients following a confirmed CeD diagnosis. The identified gaps between recommendations and clinical practice should enhance efforts to discuss implementation barriers and how ESPGHAN can further support the improvement of care.^20^ Of note, most participants reported that answering the questionnaire as a team was educational, as it exposed discrepant opinions and decision‐making among staff members and highlighted gaps in knowledge, prompting the development of standard operating procedures. Half of the participating pediatric gastroenterologists admitted that they only learned through the survey about a web‐based diagnostic app and e‐learning program(s) developed within an EU‐funded project (Focus IN CD and CD SKILLS). Consequently, the QoC task force developed pictograms with key messages about CeD in 23 languages and a checklist for physicians, available for free on the ESPGHAN website. (https://www.espghan.org/knowledge-center/education/Educational-Material-for-download).

## 5 CONCLUSION

The service evaluation in CeD diagnosis by the QoC Task Force of ESPGHAN underscores the importance of continuous monitoring and reflects the local practices and implementation of evidence‐based guidelines. When gaps are identified, targeted education and practical tools should be leveraged to enhance the translational application of current knowledge in patient‐care practice.

## CONFLICT OF INTEREST STATEMENT

The authors declare no conflicts of interest.

## Supporting information

R1 clean Litwin_CeD_supplementary files.
