# Supplementary material for: Celiac disease diagnosis in clinical practice: ESPGHAN quality of care survey from 129 pediatric hospitals across 28 countries
Source: J Pediatr Gastroenterol Nutr. 2025 Jul 7;81(3):606–17. doi: 10.1002/jpn3.70143 (PMC12408967; doi:10.1002/jpn3.70143)
Supplement: Supplementary file 1 — R1 clean Litwin_CeD_supplementary files. [file JPN3-81-606-s001.docx]

**Celiac Disease Diagnosis in Clinical Practice: ESPGHAN Quality of Care Survey from 129 Pediatric Hospitals Across 28 Countries**

Anna Litwin, Thu Giang Le Thi, Nabil El-Lababidi, Angelika Kindermann, Rouzha Pancheva, Konstantinos Gerasimidis, Cristina Campos Goncalves, Paula Crespo Escobar, Tena Niseteo, Katharina Ikrath, Sibylle Koletzko for the Quality-of-Care Network

**Supplementary tables**

[Supplementary Table 1: Serological tests for Celiac Disease diagnostic work-up applied by physicians in particular scenarios reflecting real-life clinical situations, N=129 2](#_Toc192622903)

[Supplementary Table 2: No-biopsy-diagnostic approach for CeD according to ESPGHAN guidelines’ suggestions (1) 4](#_Toc192622904)

[Supplementary Table 3: HLA-testing for diagnosis of CeD in academic and non-academic hospitals, N=129 5](#_Toc192622905)

**Supplementary figures**

[Supplementary Figure 1: Quality of Care Network of 129 hospitals in 28 European countries with completed data on Celiac disease survey (18^th^ February 2024) 6](#_Toc192622906)

[Supplementary Figure 2: Application of HLA-testing for diagnosis of CeD in different scenarios in 122 hospitals over 28 countries 7](#_Toc192622907)

**Supplementary Table 1: Serological tests for Celiac Disease diagnostic work-up applied by physicians in particular scenarios reflecting real-life clinical situations, N=129**

| **A) Which serological tests do you order for *first line* testing when CeD is suspected because of symptoms** | | | | | | | | | | |
| --- | --- | --- | --- | --- | --- | --- | --- | --- | --- | --- |
| **Test**  **Scenario** | **Total IgA** | **TGA-**  **IgA** | **TGA-IgG** | **DGP-IgA** | **DGP-IgG** | **AGA-IgA** | **AGA-IgG** | **EMA-IgA or EMA-IgG** | **Frequency** | **Proportion** |
| **1** | **●** | **●** | **-** | **-** | **-** | **-** | **-** | **-** | **85** | **66%** |
| 2 | ● | ● | - | - | - | - | - | ● | 14 | 11% |
| 3 | ● | ● | ● | - | - | - | - | - | 12 | 9% |
| 4 | - | - | - | - | - | - | - | - | 3 | 2% |
| 5 | ● | - | - | - | - | - | - | - | 3 | 2% |
| 6 | ● | ● | - | ● | - | - | - | ● | 2 | 2% |
| 7 | - | - | ● | - | - | - | - | - | 1 | <1% |
| 8 | - | ● | - | - | - | - | - | - | 1 | <1% |
| 9 | - | ● | ● | - | - | - | - | - | 1 | <1% |
| 10 | ● | ● | - | - | ● | - | - | - | 1 | <1% |
| 11 | ● | ● | - | ● | ● | - | - | ● | 1 | <1% |
| 12 | ● | ● | ● | - | - | - | - | ● | 1 | <1% |
| 13 | ● | ● | ● | - | ● | - | - | - | 1 | <1% |
| 14 | ● | ● | ● | ● | - | - | - | - | 1 | <1% |
| 15 | ● | ● | ● | ● | ● | - | - | - | 1 | <1% |
| 16 | ● | ● | ● | ● | ● | ● | ● | - | 1 | <1% |
| *We assessed data on various scenarios to determine when the following serological tests for CeD were conducted in real-life settings: TGA-IgA, TGA-IgG, DPG-IgA, DPG-IgG, AGA-IgA, AGA-IgG, EMA-IgG/IgA.*  *● indicates the test was performed/ordered.*  *- indicates the test was not performed/ordered in this specific scenario* ***or*** *not used at all at hospital.*  *Data were sorted by test frequency in descending order. We compared physicians' responses to the diagnostic work-up recommended by ESPGHAN.*  *Green bars: Correct combination according to ESPGHAN Guidelines (most effective and efficient)*  *Red bars: Unnecessary tests according to ESPGHAN Guidelines (not efficient)*  *Blue bars: Insufficient tests according to ESPGHAN Guidelines (not effective))*  *Purple bars: Possible for initial screening, but total IgA needs to be tested in a second step, since TGA-IgG positivity in the absence of IgA deficiency has a very low positive predictive value for CeD.* | | | | | | | | | | |

| **B) Which serological tests would you order for first line testing because the child is belonging to a high-risk group (e.g. child with Down-Syndrome, other autoimmune disorders like Type 1 Diabetes, thyroiditis, or having a first degree relative with CeD)** | | | | | | | | | | |
| --- | --- | --- | --- | --- | --- | --- | --- | --- | --- | --- |
| **Test**  **Scenario** | **Total IgA** | **TGA-**  **IgA** | **TGA-IgG** | **DGP-IgA** | **DGP-IgG** | **AGA-IgA** | **AGA-IgG** | **EMA-IgA or EMA-IgG** | **Frequency** | **Proportion** |
| 1 | ● | ● | - | - | - | - | - | - | 87 | 67% |
| 2 | ● | ● | - | - | - | - | - | ● | 14 | 11% |
| 3 | ● | ● | ● | - | - | - | - | - | 10 | 8% |
| 4 | - | - | - | - | - | - | - | - | 4 | 3% |
| 5 | ● | - | - | - | - | - | - | - | 3 | 2% |
| 6 | ● | ● | ● | - | - | - | - | ● | 2 | 2% |
| 7 | - | - | ● | - | - | - | - | - | 1 | <1% |
| 8 | - | ● | - | - | - | - | - | - | 1 | <1% |
| 9 | ● | ● | - | - | ● | - | - | - | 1 | <1% |
| 10 | ● | ● | - | ● | - | - | - | ● | 1 | <1% |
| 11 | ● | ● | - | ● | ● | - | - | ● | 1 | <1% |
| 12 | ● | ● | ● | - | ● | - | - | - | 1 | <1% |
| 13 | ● | ● | ● | ● | - | - | - | - | 1 | <1% |
| 14 | ● | ● | ● | ● | ● | - | - | - | 1 | <1% |
| 15 | ● | ● | ● | ● | ● | ● | ● | - | 1 | <1% |
| *We assessed data on various scenarios to determine when the following serological tests for CeD were conducted in real-life settings: TGA-IgA, TGA-IgG, DPG-IgA, DPG-IgG, AGA-IgA, AGA-IgG, EMA-IgG/IgA.*  *● indicates the test was performed/ordered.*  *- indicates the test was not performed/ordered in this specific scenario* ***or*** *not used at all at hospital.*  *Data were sorted by test frequency in descending order. We compared physicians' responses to the diagnostic work-up recommended by ESPGHAN. Unnecessary tests (in grey bar) were identified as those routinely ordered by physicians but not mentioned in ESPGHAN guidelines.*  *Green bars: Correct combination according to ESPGHAN Guidelines (most effective and efficient)*  *Red bars: Unnecessary tests according to ESPGHAN Guidelines (not efficient)*  *Blue bars: Insufficient tests according to ESPGHAN Guidelines (not effective)* | | | | | | | | | | |

| **C) Second-line test for confirmatory serology for the non-biopsy diagnosis if first TGA-IgA was >10 times upper the limit of normal** | | | | | | | | | |
| --- | --- | --- | --- | --- | --- | --- | --- | --- | --- |
| **Test**  **Scenario** | **TGA-**  **IgA** | **TGA-IgG** | **DGP-IgA** | **DGP-IgG** | **AGA-IgA** | **AGA-IgG** | **EMA-IgA or EMA-IgG** | **Frequency** | **Proportion** |
| **1** | ● | - | - | - | - | - | ● | 61 | 47% |
| **2** | - | - | - | - | - | - | ● | 32 | 25% |
| 3 | - | - | - | - | - | - | - | 17 | 13% |
| 4 | ● | - | - | - | - | - | - | 7 | 5% |
| 5 | ● | ● | - | - | - | - | ● | 5 | 4% |
| 6 | - | - | ● | - | - | - | - | 2 | 2% |
| 7 | - | ● | - | - | - | - | ● | 1 | <1% |
| 8 | ● | - | - | ● | - | - | ● | 1 | <1% |
| 9 | ● | - | ● | - | - | - | ● | 1 | <1% |
| 10 | ● | - | ● | - | ● | - | ● | 1 | <1% |
| 11 | ● | ● | ● | ● | - | - | ● | 1 | <1% |
| *We assessed data on various scenarios to determine when the following serological tests for CeD were conducted in real-life settings: TGA-IgA, TGA-IgG, DPG-IgA, DPG-IgG, AGA-IgA, AGA-IgG, EMA-IgG/IgA.*  *● indicates the test was performed/ordered.*  *- indicates the test was not performed/ordered in this specific scenario* ***or*** *not used at all at hospital.*  *Data were sorted by test frequency in descending order. We compared physicians' responses to the diagnostic work-up recommended by ESPGHAN. Unnecessary tests (in grey bar) were identified as those routinely ordered by physicians but not mentioned in ESPGHAN guidelines.*  *Green bars: Correct combination according to ESPGHAN Guidelines (most effective and efficient)*  *Red bars: Unnecessary tests according to ESPGHAN Guidelines (effective, but not efficient)*  *Blue bars: Insufficient tests according to ESPGHAN Guidelines (not effective)* | | | | | | | | | |

**Supplementary Table 2: No-biopsy-diagnostic approach for CeD according to ESPGHAN guidelines’ suggestions (1)**

| **Factors n (%)** | **Total** | **Academic hospitals** | **Non-academic hospitals** | **P-value ^b)^** |
| --- | --- | --- | --- | --- |
| **Non*-*biopsy-diagnosis for CeD is *not* used, N=129** | 15 (12%) | 9 (11%) | 6 (14%) | 0.61 |
| **What is the main reason for not choosing the non-biopsy diagnosis in selected patients? N=15** | | | | |
| I find the data not convincing, and I am afraid to make a false  positive diagnosis | 3 | 3 | 0 | n.a. |
| I find it more difficult to convince the parents, that their child has CeD at time of diagnosis or after it started the GFD | 7 | 5 | 2 | n.a. |
| For research purposes it is valuable to have the biopsies | 4 | 4 | 0 | n.a. |
| Our hospital/institution is not able to perform EMA testing and sending it to an outside lab is too costly or not covered by insurance | 6 | 3 | 3 | n.a. |
| EMA testing is not available in our country | 2 | 2 | 0 | n.a. |
| Reimbursement difficulties / Insurance requires biopsy | 3 | 3 | 0 | n.a. |
| **Non-biopsy-diagnosis for CeD in selected patients as suggested by the ESPGHAN guidelines (1) is used, N=129** | 114 (88%) | 76 (89%) | 38 (86%) | 0.61 |
| **In your current practice, which of the following conditions/findings should be present for CeD diagnosis without biopsies? N=114** | | | | |
| Symptoms of malabsorption | 57 (50%) | 38 (50%) | 19 (50%) | 1.0 |
| Any symptoms | 52 (46%) | 37 (49%) | 15 (39%) | 0.07 |
| Absence of type-1 diabetes mellitus | 48 (42%) | 31 (41%) | 17 (45%) | 0.15 |
| Absence of IgA deficiency | 77 (68%) | 50 (66%) | 27 (71%) | 0.03 |
| TGA-IgA >10xULN performed in- or outside our hospital | 110 (96%) | 73 (96%) | 37 (97%) | 0.17 |
| TGA-IgA even <10xULN (e.g. 8xULN) because we use a very specific test | 3 (3%) | 3 (4%) | 0 (0%) | 0.06 |
| Use of a TGA-IgA test which has been validated and published for the use of non-biopsy CeD diagnosis | 56 (49%) | 42 (55%) | 14 (37%) | 0.15 |
| CeD autoimmunity confirmed in a second blood sample by positive EMA | 100 (88%) | 65 (86%) | 35 (92%) | 0.10 |
| CeD autoimmunity confirmed in a second blood sample by positive TGA-IgA or DGP-IgG (as substitute for EMA) | 34 (30%) | 18 (24%) | 16 (42%) | 0.01 |
| Tests showing HLA DQ2 and/or DQ8 positivity | 13 (11%) | 10 (13%) | 3 (8%) | 0.27 |
| In children with TGA-IgA >10xULN, caregiver/patients are always informed prior to taking the second blood sample (if not done outside) on the PROs and CONs of the diagnosis with or without biopsies by a paediatric gastroenterologist | 98 (86%) | 66 (87%) | 32 (84%) | 0.36 |
| In certain situations, we try to convince caregiver to perform biopsies (although all prerequisites for the no-biopsy approach are fulfilled) | 38 (33%) | 24 (32%) | 14 (37%) | 0.10 |
| **How often did you use the no-biopsy diagnosis during the last 12 months in children having TGA-IgA >10xULN? N=114** |  |  |  | 0.72 |
| >80% | 50 (44%) | 33 (43%) | 17 (45%) |  |
| 50-80% | 31 (27%) | 23 (30%) | 8 (21%) |  |
| 20-50% | 23 (20%) | 15 (20%) | 8 (21%) |  |
| <20% | 6 (5%) | 3 (4%) | 3 (8%) |  |
| I do not know | 4 (4%) | 2 (3%) | 2 (5%) |  |
| *a) Academic hospitals include university hospitals, while non-academic hospitals encompass non-university public paediatric hospitals, non-academic public general hospitals with paediatric departments or divisions, church or charity‐owned hospitals and other similar non-university institutions.*  *b) P-value obtained by Pearson’s Chi-square test to determine a significant difference in pre-conditions in academic and non-academic hospitals. Bold p-values indicate significant differences in the proportion of respective factors between comorbidity groups with a p-value ≤0.05.*  *c) The test is performed outside of the hospital or institution, i.e., in another hospital or commercial lab.*  ***Abbreviations:*** *CeD - celiac disease, GI- gastrointestinal, IgA - Immunoglobulin A, IgG - Immunoglobulin G, TGA- transglutaminase antibodies, DGP- deaminated gliadin peptides, EMA- endomysia antibodies, ULN - Upper limit of normal*  *Green bars: These items should be present to for the option to diagnose CeD without biopsies according to ESPGHAN Guidelines*  *Blue bar: There are insufficient data in children having type 1 diabetes, therefore no clear ESPGHAN recommendations* | | | | |

**Supplementary Table 3: HLA-testing for diagnosis of CeD in academic and non-academic hospitals, N=129**

| **Factors n (%)** | **Total**  **N=129** | **Academic hospitals**  **N=85 (66%)** | **Non-academic hospitals**  **N=44 (34%)** | **P-value ^b)^** |
| --- | --- | --- | --- | --- |
| **Do you have access to determination of HLA CeD risk alleles (HLA DQ2 and DQ8)** | | | | **<0.01** |
| Yes, I can order it without any justification | 87 (68%) | 62 (73%) | 25 (57%) |  |
| Yes, I can order for any patient, but not in healthy persons | 5 (4%) | 1 (1%) | 4 (9%) |  |
| Yes, I can order it but only for patients in certain situation | 19 (15%) | 12 (14%) | 7 (16%) |  |
| Yes, but I have to refer the patient to a geneticist for testing | 3 (2%) | 3 (4%) | 0 (0%) |  |
| Yes, but patient has to generate the results in a private lab | 8 (6%) | 6 (7%) | 2 (5%) |  |
| No access at all | 7 (5%) | 1 (1%) | 6 (13%) |  |
| **Do you know what method the laboratory you collaborate uses for HLA DQ2 and DQ8, N=121** | | | | 0.17 |
| Yes, a commercial test covering the frequent risk alleles (e.g., DQ 2.5, DQ 2.2, DQ8, DQ7.5) | 43 (35%) | 26 (33%) | 17 (41%) |  |
| Yes, a commercial test covering also rare risk alleles (e.g., DQ9, DQ2.3) | 7 (6%) | 7 (9%) | 0 (0%) |  |
| Yes, an in house made test covering the frequent risk | 7 (6%) | 4 (5%) | 3 (7%) |  |
| Yes, an in house made test covering also rare risk alleles | 6 (5%) | 5 (6%) | 1 (2%) |  |
| Yes, always sequencing | 3 (3%) | 2 (3%) | 1 (2%) |  |
| Other | 2 (1%) | 0 (0%) | 2 (5%) |  |
| I do not know | 53 (44%) | 35 (44%) | 18 (43%) |  |
| *a) Academic hospitals include university hospitals, while non-academic hospitals encompass non-university public paediatric hospitals, non-academic public general hospitals with paediatric departments or divisions, church or charity‐owned hospitals and other similar non-university institutions.*  *b) P-value obtained by Pearson’s Chi-square test to determine a significant difference in pre-conditions in academic and non-academic hospitals. Bold p-values indicate significant differences in the proportion of respective factors between comorbidity groups with a p-value ≤0.05.*  *Abbreviations: CeD, Celiac disease* | | | | |

**
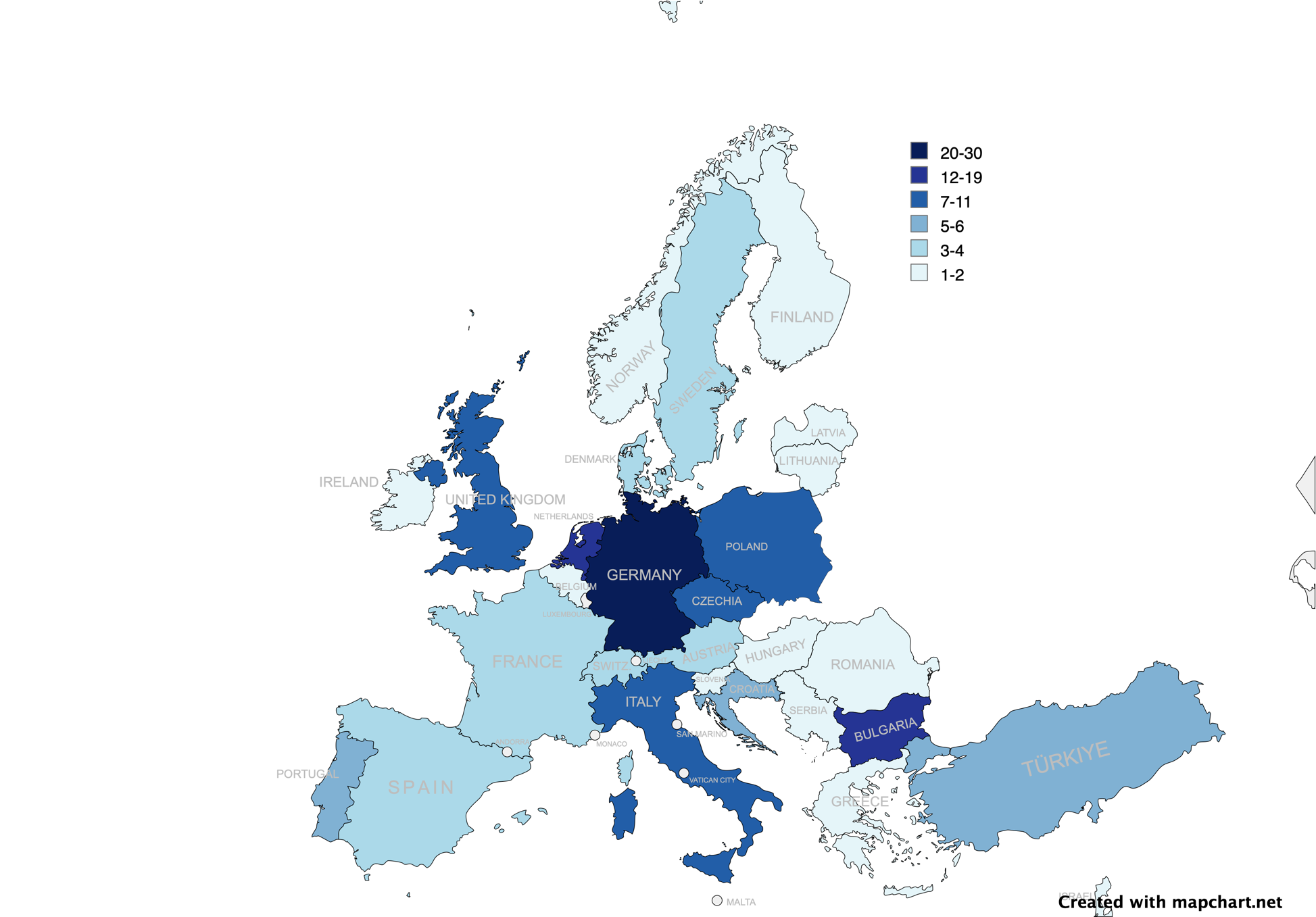
**

**Supplementary Figure 1: Quality of Care Network of 129 hospitals in 28 European countries with completed data on Celiac disease survey (18^th^ February 2024)**

**Supplementary Figure 2: Application of HLA-testing for diagnosis of CeD in different scenarios in 122 hospitals over 28 countries**

***Abbreviations:*** *CeD - celiac disease, GI- gastrointestinal, IgA - Immunoglobulin A, IgG - Immunoglobulin G, TGA- transglutaminase antibodies, GFD, gluten free diet*

**Reference**

1. Husby S, Koletzko S, Korponay-Szabó I, Kurppa K, Mearin ML, Ribes-Koninckx C, et al. European Society Paediatric Gastroenterology, Hepatology and Nutrition Guidelines for Diagnosing Coeliac Disease 2020. J Pediatr Gastroenterol Nutr. 2020;70(1):141-56.
